# Prescribing patterns before the initiation of novel antidiabetic medicines in public, occupational, and private healthcare: a register study reflecting the guidelines of care in type 2 diabetes

**DOI:** 10.1186/s12913-024-12010-y

**Published:** 2024-12-05

**Authors:** Hanna Rättö, Mikko Nurminen, Katri Aaltonen

**Affiliations:** 1 Research Unit, The Social Insurance Institution of Finland, Helsinki, Finland; 2 INVEST Research Flagship Centre, University of Turku, Turku, Finland

**Keywords:** Diabetes, Healthcare, Novel medicine, Care guideline

## Abstract

**Background:**

Disparities in access to healthcare has been implied before in Finland, a country with universal healthcare but *de facto* tiered primary care. Less is however known about the content of care provided in different settings. Previous studies indicate potential disparities in prescribing newer medicines between healthcare sectors. We compared the preceding prescribing patterns of patients who initiated a sodium-glucose co-transporter 2 (SGLT2) inhibitor or a glucagon-like peptide-1 (GLP-1) analogue in public, occupational, and private healthcare.

**Methods:**

We used logistic models and patient-level register data from the city of Oulu, Finland, during 2014–2018. Among patients who initiated SGLT2 inhibitors or GLP-1 analogues, we studied whether it was a first-line treatment or if other antidiabetic medicines preceded the use. In addition, prior use of statins (a lipid-lowering medicine) and insulins were studied. Clinical guidelines for type 2 diabetes recommend in most cases metformin in first-line, and insulin only at later stages or in case of severe hyperglycaemia. Using a lipid-lowering medicine is typically recommended for all.

**Results:**

The examined novel antidiabetic medicines were seldom initiated in first-line, and no significant differences were observed for preceding statin use across sectors, net of patient characteristics. However, patients in the public sector were more likely to have used insulin previously compared to patients in occupational sector.

**Conclusions:**

Before the initiation of the examined novel antidiabetic medicines, no marked differences across sectors in the use of other antidiabetic medicines or statins were observed. The higher likelihood of prior insulin use in the public sector might reflect initiation at a later stage and/or unobserved differences in clinical characteristics across patient populations.

**Supplementary Information:**

The online version contains supplementary material available at 10.1186/s12913-024-12010-y.

## Introduction

Diabetes is one of the key public health priorities among non-communicable diseases and has even been called ‘a defining disease of the 21st century’ [1, 2]. The burden of diabetes is inequitably distributed, as socioeconomic position has been associated with prevalence, disease progression and complication risk in diabetes [2–7]. Globally type 2 diabetes is estimated to have accounted for 90% of all diabetes prevalence in 2021 [8]. It is associated with microvascular- and macrovascular complications, and cardiovascular complications are a marked factor in diabetes mortality [9–12]. Thus, in addition to balanced glycaemic control, maintaining low blood pressure and cholesterol level are important parts of comprehensive care of type 2 diabetes.

Treatment of type 2 diabetes has gone through several therapeutic shifts in the past decades with subsequent market entries of novel medicines [13–16]. In addition to the glucose-lowering effects of the novel medicines, accumulating clinical evidence suggests cardiovascular and renal benefits for those at high -risk [10, 17]. Nevertheless, newer medicines tend to enter the market with increasingly high prices, putting pressure on the health budgets. In Finland, the therapeutic shifts to using newer blood-glucose lowering medications contributed considerably to the rapid growth of the outpatient pharmaceutical reimbursement expenditure in the 2010s [13]. If prescribing patterns are inappropriate, they can result in increased pharmaceutical expenditure with little or no added health benefits [18–21].

In Finland, the Current Care Guidelines for Type 2 diabetes outlines the principles of pharmacotherapy [22]. The guidelines are independent, evidence-based, and are developed by the Finnish Medical Society Duodecim in association with medical specialist associations [23]. Development is supported by systematic literature searches, and guidelines are updated regularly. Considerations of cost-effectiveness are not included in Finnish clinical guidelines [24]. Besides guidelines, prescribing is conditioned by the national reimbursement system [25]. (See also Supplementary file 1.)

Diabetes is historically divided into type 1 and 2, although the condition is better described as a spectrum. Type 1 diabetes is characterized by insulin deficiency, which is compensated by insulin pharmacotherapy. Type 2 diabetes is, in turn, characterised by insulin resistance, although insulin deficiency may develop over time as the disease progresses. Accordingly, type 2 diabetes is a heterogeneous disease requiring person-centered care that accounts for individual differences in, e.g., onset age, insulin resistance, obesity, and complication tendency [10]. Insulin pharmacotherapy is associated with an increased risk for hypoglycaemia and weight gain; thus, its use in type 2 diabetes is typically recommended in later stages, when hyperglycaemia cannot be adequately controlled with other treatments, or when they are contraindicated [26].

During the study period, the 2013 version of the Finnish Current Care Guideline [27] recommended in most cases metformin in first line. In second line, several options were recommended, e.g., sulfonylureas, glinides, thiazolidinediones (glitazones), dipeptidyl peptidase 4 (DPP-4) inhibitors, GLP-1-analogues, and SGLT2-inhibitors. Metformin with or without DPP-4 inhibitors, GLP-1-analogues, glitazones, or SGLT2-inhibitors were mentioned as options with no risk of hypoglycemia. The 2018 version [28] further highlighted individualised pharmacotherapy, accounting for patient’s self-management, hypoglycemia risk, stage of the disease, comorbidities, and life expectancy. People with diabetes are considered at risk for arterial disease, to whom lipid-lowering medications are typically recommended (statins in first-line) [29]. Prescribing of lipid-lowering medications in diabetes is also used as an indicator of the quality of prescribing in primary care in OECD’s collection of health-related indicators [30]. 

Primary care has an important role in the monitoring and management of diabetes [22]. The use of primary care in Finland has demonstrated relatively large socioeconomic disparities, commonly attributed to the *de facto* tiered primary care system where public, occupational and private options are available [31–34]. Public healthcare provides services for all residents. The occupational sector serves the employed working-age population. The oldest and those outside the labour force predominantly use the public sector. Compared to the public sector, occupational healthcare typically has shorter waiting times and is free at the point of service. Private healthcare is accessible to those who can afford to pay most of the costs out-of-pocket. Waiting times to private care are also often short, but the availability of services can differ between regions. Thus, the use of occupational and private care is skewed towards the better-off population groups [34–37]. (See also Supplementary file 1.) Beyond differences in access [38], differences in the quality and content of services between healthcare sectors might apply. Factors not necessarily related to need, such as the patient’s socioeconomic position, the physician’s characteristics or the service provider’s internal decision making might also be realised in the differences in the use of services [21, 39, 40].

National guidelines regarding prescription medicines apply similarly to prescribers across healthcare sectors, and medicines used in outpatient setting are reimbursed similarly from the National Health Insurance (NHI) regardless of the sector where the prescriber operates. Further, prescribers are not differentially financially rewarded for prescribing certain pharmaceutical products over others. Nevertheless, available studies have suggested disparities in the prescribing patterns of newer medicines between healthcare sectors, with patients in public healthcare being less likely to receive prescriptions for newer medicines than patients in other sectors [41, 42]. These results parallel findings suggesting that patients with higher socioeconomic status are more likely to initiate newer pharmacological treatments [43–45]. These findings extend to antihyperglycemic and cardiovascular medicines [46–48]. In addition to new treatments, socioeconomic differences have in other settings also been implied in the patterns of guideline-recommended medication use [49].

The aim of this study was to compare whether the use of SGLT2 inhibitors or GLP-1 analogues in first-line differed between public, occupational and private healthcare in Finland. Using regional register data between 2014 and 2018, we studied the preceding medications used by patients who initiated SGLT2 inhibitor or GLP-1 analogue pharmacotherapy. In more detail, we examined whether the individuals had been dispensed other medications belonging to the Anatomic Therapeutic Classification (ATC; 51) category A10B (Blood glucose lowering drugs, excl. insulins, hereafter: antidiabetic medicines), insulin and analogues (ATC A10A, hereafter: insulins), and HMG CoA reductase inhibitors (ATC C10AA, hereafter: statins).

## Materials and methods

### Medicine reimbursements in Finland

All permanent residents are entitled to *medicine reimbursements* for outpatient prescription medicines reimbursable from the NHI. The universal basic reimbursement rate is 40% of the retail price. In addition, two categories for special reimbursements exist. Special reimbursements are eligibilities to higher reimbursement based on diagnosed severe or chronic diseases requiring outpatient pharmacotherapy, such as cancers, asthma, rheumatoid arthritis, hypertension, coronary heart disease (CHD), or diabetes [50]. SGLT2 inhibitors and GLP-1 analogues were first reimbursed in 2013 (special reimbursement in 2016) and in 2011 (special reimbursement in 2013), respectively. GLP-1 analogues are reimbursed restrictedly, meaning that patients need to fulfill specific clinical criteria to be granted eligibility.

## Data and measures

### Data sources

Data were collected from several registers on the residents of Oulu, the fifth largest city in Finland. Finland has approximately 5.5 million residents from which around 200,000 live in Oulu. The register data used in the study have been described in more detail on a report by Blomgren & Jäppinen [51]. In 2018, approximately 10 000 individuals in Oulu had an entitlement for special reimbursements based on diabetes [52].

From patient-level register -data, we selected all patients who had initiated SGLT2 inhibitor or GLP-1 analogue for the first time between 2014 and 2018 in Oulu. Data on prescription medicine purchases reimbursed under the NHI were collected from the Dispensations reimbursable under the NHI scheme -register maintained by the Social Insurance Institution of Finland (Kela). In addition to the pseudonymised patient identifier and purchase date, the pseudonymised identifier of the prescribing physician and prescribing date are included in the register. Information on healthcare service use in public setting was collected from the registers of Oulu and the Care Registers for Health Care maintained by The Finnish Institute for Health and Welfare. Information on the use of occupational healthcare was collected from the patient registers of four large occupational healthcare providers in the Oulu area. Together these registers cover over 90% of the visits to occupational healthcare in Oulu [53]. Information on the use of private healthcare was collected from the register maintained by Kela. The register includes data on all visits to private healthcare for which public reimbursement from NHI has been received. Among other things, information on the patient, the physician and the date of the visit are recorded in the register [54].

Information on demographic variables (age, sex) and comorbidities (measured using special reimbursement entitlements) were collected from registers maintained by Kela. Annual-level information on taxable income were collected from the Finnish Tax Administration.

### People with type 2 diabetes

We used individual-level register data from 2013 to 2018 to study patients who initiated SGLT2 inhibitor or GLP-1 analogue between 2014 and 2018 in public, occupational and private healthcare. We identified these treatment initiators based on a reimbursed purchase of a medicinal product belonging to ATC [55] classification category A10BJ (GLP-1 analogues) or A10BK (SGLT2 inhibitors), or of a combination product (ATC category A10BD) that included a SGLT2-inhibitor, and no preceding purchases of these medications in the preceding 365 days. We defined the prescribing date of the first SGLT2 inhibitor or GLP-1 analogue as the date of initiation.

### Identifying the prescribing healthcare sector

Since data on reimbursed medicine purchases does not contain information on the healthcare sector where the medicine was prescribed (public, private or occupational), we adapted a method described in a report by Miettinen et al. [56], shortly described below.

We linked the data on the patient’s first purchase of a SGLT2 inhibitor or GLP-1 analogue to data on healthcare visits. From the first purchase, we extracted information on the pseudonymised identifier of patient and the prescribing physician, as well as the date the medicine was prescribed. From the registers of healthcare visits in public and private healthcare, we extracted information on the pseudonymised identifiers of the patient and the physician, as well as the date of the visit.

First, we linked prescriptions to visits in public and private sector using the pseudonymised patient and prescribing physician identifiers, and prescription and visit dates. Second, we linked the prescriptions to occupational healthcare using the patient identifier and the date of prescribing/visit, since the pseudonymised identifier of physician was not available in occupational healthcare. Finally, the remaining prescriptions were allocated by prioritising the public sector over the others and occupational sector over private sector (for more detailed description of the process, see Supplementary file 2).

### Outcome variables and covariates

We used three different outcomes to measure prior medication use (365 days before initiation): use of another antidiabetic medicine, insulin and statin. Prior use of these medicines was examined based on whether the individual had at least one reimbursed purchase.

Several covariates were constructed to measure the individuals’ morbidity and socioeconomic and demographic background. We used two binary variables to measure morbidity: diabetes-related hospital contacts and comorbidities defined based on special reimbursement entitlements. We defined the former as whether an individual had at least one outpatient or inpatient stay in hospital-setting with the primary diagnosis related to diabetes (ICD-10 codes E10-E14) as the primary diagnosis during 365 days preceding the initiation of the novel non-insulin antidiabetic medicine. The latter was defined as whether an individual had at least one entitlement to special reimbursement for medicine expenses (excluding the possible entitlement for diabetes) at the beginning of the year of initiation. Special reimbursement codes are commonly used as proxies of morbidity in studies based on Finnish health registers [57, 58]. Socioeconomic and demographic covariates included age, sex, and taxable income at the end of the year. Consumer price index was used to harmonise the income-levels across the years.

### Statistical methods

We used logistic regression models to compare the preceding use of antidiabetic medicines and statins in public, occupation, and private healthcare. All three outcomes were estimated from a separate model. Furthermore, we estimated unadjusted models and models adjusted for the covariates. We reported the odds ratios (OR) of the estimates with their 95% confidence intervals (CI). We also grouped patients to two groups: the group including all patients irrespective of their age, and the group including only those aged 25–64 years. This was done because the occupational healthcare is in prominent position among the working-age patients [37, 59].

## Results

A total of 2,319 individuals initiated the use of a SGLT2 inhibitor or GLP-1 analogue in Oulu during 2014–2018. Most of them (76%) received the prescription from public healthcare (Table 1). For approximately 18%, the prescription originated from occupational healthcare and for 6% private healthcare. Almost 60% of initiators (1,337 patients) were working-age (25–64 years), and of them, approximately 64% received the prescription from public healthcare, 31% from occupational healthcare and 5% from private healthcare.

**Table 1:** Characteristics of patients initiating SGLT2 inhibitor or GLP-1 analogue with prescriptions from different healthcare sectors 2014–2018

	All initiators			Working-age (25–64) initiators		
	Public healthcare	Occupational healthcare	Private healthcare	Public healthcare	Occupational healthcare	Private healthcare
Number of patients	1754	422	143	855	412	70
Mean age (years)	63	54	64	53	54	55
Median taxable income (2017 prices)	€20 176	€42 303	€31 079	€19 238	€41 527	€41 681
Number (share) of men	981 (55.9%)	264 (62.6%)	82 (57.3%)	511 (59.8%)	257 (62.4%)	46 (65.7%)
Number (share) of patients with hospital-level specialist healthcare use the previous year	990 (56.4%)	161 (38.2%)	69(48.3%)	444 (51.9%)	156 (37.9%)	28 (40.0%)
Number (share) of patients with diabetes-related, hospital-level specialist healthcare use the previous year	151 (8.6%)	< 10 (< 2.3%)	< 10(< 7%)	75 (8.8%)	< 10 (< 2.4%)	< 10 (< 14.2%)
Number (share) of patients with comorbidity^a^	1 296 (73.9%)	244 (57.8%)	100 (69.9%)	532 (62.2%)	240 (58.3%)	44 (62.9%)
Number (share) of patients with prior antidiabetic medicine use	1 648 (94.0%)	400 (94.8%)	128 (89.5%)	804 (94.0%)	390 (94.7%)	63 (90.0%)
Number (share) of patients with prior statin	1 157 (66.0%)	218 (51.6%)	86 (60.1%)	493 (57.7%)	213 (51.7%)	38 (54.3%)
Number (share) of patients with prior insulin	600 (34.2%)	54 (12.8%)	39 (27.3%)	299 (35.0%)	53 (12.9%)	17(24.3%)

^a^Defined based on special reimbursement entitlement

Results comparing the prior use of antidiabetic medicines, statins, and insulins across sectors using logistic models are presented in Table 2. In all models, comparisons across sectors are presented using ORs and their 95% CIs, with public healthcare used as a reference sector. Further, as the reimbursements related to antidiabetic medicines underwent several changes during the study period, we also controlled for the year of the initiation (see Supplementary file 3 for full models).

**Table 2:** Associations between healthcare sector and prior use of non-insulin antidiabetic medicines, insulins and statins

	Unadjusted models		Models adjusted for patient characteristicst^a^		Models adjusted for patient characteristics and the year of the initiation^b^	
	OR	95% CI	OR	95% CI	OR	95% CI
**All initiators**						
* Model 1: Prior use of another non-insulin antidiabetic medicine*						
Public healthcare	1		1		1	
Occupational healthcare	1.17	0.73–1.88	1.27	0.74–2.20	1.25	0.72–2.17
Private healthcare	0.55**	0.31–0.97	0.56	0.31–1.01	0.51**	0.28–0.93
* Model 2: Prior use of statin*						
Public healthcare	1		1			
Occupational healthcare	0.55***	0.45–0.68	0.82	0.63–1.06	0.82	0.63–1.06
Private healthcare	0.78	0.55–1.10	0.76	0.53–1.10	0.74	0.51–1.08
* Model 3: Prior use of insulin*						
Public healthcare	1		1			
Occupational healthcare	0.28***	0.21–0.38	0.37***	0.26–0.52	0.35***	0.24–0.49
Private healthcare	0.72	0.49–1.06	0.84	0.56–1.25	0.76	0.50–1.13
**Working-age initiators**						
* Model 1: Prior use of another antidiabetic medicine*						
Public healthcare	1		1			
Occupational healthcare	1.12	0.67–1.88	1.12	0.61–2.06	1.10	0.60–2.03
Private healthcare	0.57	0.25–1.31	0.53	0.22–1.29	0.48	0.2–1.17
* Model 2: Prior use of statin*						
Public healthcare	1		1			
Occupational healthcare	0.79**	0.62–1.00	0.78	0.58–1.05	0.78	0.58–1.05
Private healthcare	0.87	0.53–1.42	0.78	0.46–1.32	0.76	0.45–1.29
* Model 3: Prior use of insulin*						
Public healthcare	1		1			
Occupational healthcare	0.28***	0.20–0.38	0.37***	0.26–0.54	0.33***	0.23–0.49
Private healthcare	0.60	0.34–1.05	0.72	0.40–1.31	0.58	0.31–1.07

^a^Adjusted for age, sex, comorbidities, diabetes-related hospital contact, taxable income

^b^Adjusted for age, sex, comorbidities, diabetes-related hospital contact, taxable income, the year of initiation

**p < 0.05

***p<0.0001

Results for initiators of all ages and of working-age largely parallel each other. The findings suggest that, when accounting for patient characteristics, there was no statistically significant difference between sectors in the odds of having used another antidiabetic medicine before initiating a novel antidiabetic medicine. When controlling also for the initiation year, the OR for private healthcare became statistically significant but the magnitude of the point estimate remained intact. Regarding prior use of statins and insulins, results suggest lower odds of prior use in initiators in occupational healthcare compared to the initiators in public healthcare. However, after taking patient characteristics into account, the association regarding statin use loses statistical significance. The odds for prior insulin use remains significantly smaller among initiators in occupational healthcare than those in the public healthcare.

## Discussion

We compared the prescribing patterns through prior use of other antidiabetic medicines, statins and insulin in patients initiating a SGLT2 inhibitor or GLP-1 analogue in public, occupational, and private healthcare in Finland. We found that the use of SGLT2 inhibitors or GLP-1 analogues in first-line was rare in all sectors, with over 90% of initiators having preceding use of another type of antidiabetic medicine. After accounting for patient characteristics, no significant differences between sectors were found in prior statin use. Prior insulin use was not significantly different in public and private care; however, initiators in public sector were more likely to have used insulin than initiators in occupational care.

Differences in the characteristics of initiators across sectors reflected the known socioeconomic distribution of patient populations in different healthcare sectors: on average initiators in public healthcare had lower income than patients initiating with prescriptions from the other sectors. Patients in public healthcare were also more likely to have used hospital-level specialist care, suggesting higher morbidity.

The results suggest compliance with national clinical guidelines of care regardless of sector. For GLP-1 analogues, the reimbursement restriction based on clinical criteria may have also played a role. Results of statin use align with previous findings in Finland [30]. However, prior insulin use was more common in the public sector than in the occupational sector. This may reflect differences across patient populations in disease stage, insulin deficiency and co-morbidities, such as, morbid obesity [60], which may be due to distribution of patients across sectors based on socioeconomic determinants [34, 37].

However, a tendency to prescribe SGLT2 inhibitor or GLP-1 analogue at a later stage in public setting might also play a role. Despite the fact that out-of-pocket costs of prescription medicines do not depend on the sector in which they are prescribed, there may be still be both demand and supply side factors that contribute to potential differences between sectors. Patients in private sector potentially may demand newer medicines because of an impression of better quality. On the supply side, physicians’ knowledge of newer medicines and willingness to satisfy patients’ medicine choice preferences may differ between the sectors. Previous studies have suggested disparities between healthcare sectors in prescribing newer medicines, including antidiabetics [41, 42]. Our findings concentrating on prescribing patterns prior to the initiation of SGLT2 inhibitor or GLP-1 analogue and reflecting clinical guidelines at initiation add to these results. As novel antidiabetic medicines are an important example of the increasing gap between the technological and fiscal possibilities reflecting the sustainability challenges all health systems face today, their uptake should be monitored to ensure appropriate use.

A majority of prescriptions for SGLT2 inhibitors or GLP-1 analogues were issued in public healthcare, however, occupational healthcare also had a prominent role. Our results thus align with previous findings of occupational care being an important service provider for the working-age population [34, 37] also in prescribing medicines, especially for conditions typically treated in primary care [41, 59]. Our results also further demonstrate that medicines for chronic conditions requiring long-term treatment and monitoring are prescribed in all sectors of primary care. The entitlement for occupational healthcare in Finland is typically tied to employment. Thus, attention should be paid to ensuring the continuity of pharmacotherapy, when conditions related to employment change.

The strengths of our study include rich and comprehensive register data that also included data on occupational healthcare utilisation, which has scarcely been available for research before. As features such as privatisation and public-private mixes are increasingly present in all health systems, our findings add to the on-going discussion on their implications. Furthermore, being able to study the initiation of novel antidiabetic medicines is a considerable strength, as the prescriber has more agency in initiation than in renewing existing prescriptions.

However, the study also has several limitations. First, potential variation between regions may decrease the external validity of the results to the national level. However, despite some variation in living conditions may exist, city of Oulu does not differ in any systemic way in terms of demographic, socioeconomic, or healthcare utilisation related factors from the average Finnish population [51]. Second, we were unable to match some of the prescriptions to their respective healthcare sectors using solely patient and physician identifiers and dates of service use. Thus, there is a possibility of some prescriptions being linked to wrong sectors. Third, our data are based on reimbursed medicine purchases. Therefore, implications on non-reimbursed prescription medicines or prescriptions that were never dispensed cannot be made. Fourth, assessing the optimal timing of the treatment intensification is beyond the scope of the current study, as the data do not contain information on clinical characteristics, such as glycemic control or the weight of the patient. Finally, as our findings are of descriptive nature, future studies should examine the causal effects of introducing new diabetes medicines to the market, including effects in different healthcare sectors.

## Conclusions

No marked differences across sectors in examined prescribing patterns regarding prior use of antidiabetic medicines or statins were observed. SGLT2 inhibitors or GLP-1 analogues were seldom initiated in first-line. However, patients in the public sector were more likely to have previously used insulin than patients in occupational care, suggesting initiation at a later stage and/or unobserved differences in clinical characteristics across patient populations.

## Supplementary Information

Supplementary Material 1.

Supplementary Material 2.

Supplementary Material 3.

## Data Availability

Due to legal restrictions and data protection regulations of the administrative sources providing individual-level register data, the authors do not have the permission to make sensitive personal data available. Concerning data on health services of the City of Oulu, the occupational healthcare providers, National Institute for Health and Welfare, and data of the Social Insurance Institution of Finland, interested parties may apply for permissions to access the data from the centralised data permit authority Findata (https://www.findata.fi/en/) at email: info@findata.fi, tel: +358295246500. Data on taxable income may be applied from the Finnish Tax Administration, verohallinto@vero.fi, P.O. Box 325, 00052 VERO.
