# Supplementary material for: Prescribing patterns before the initiation of novel antidiabetic medicines in public, occupational, and private healthcare: a register study reflecting the guidelines of care in type 2 diabetes
Source: BMC Health Serv Res. 2024 Dec 5;24:1553. doi: 10.1186/s12913-024-12010-y (PMC11619279; doi:10.1186/s12913-024-12010-y)
Supplement: Supplementary file 1 — Supplementary Material 1. [file 12913_2024_12010_MOESM1_ESM.pdf]

Supplementary file 1.

The number references refer to the references in this Supplementary file.

The Finnish system for healthcare and medicine reimbursements

Public healthcare consists of mainly tax-funded (a small user fee usually applies [1, 2, 3] and publicly provided comprehensive services from primary to tertiary care to all residents. Hospital services and highly specialised outpatient care are predominantly provided in the public sector. Nurses and physicians act as gatekeepers to these services. Municipalities were responsible for organising public healthcare services until 2023, when the responsibility was shifted to larger regions (wellbeing services counties) [4].

Occupational healthcare includes primary and sometimes specialist medical care, and covers 89% of all employees (corresponding to 35% of the entire Finnish population in 2020). Employers purchase occupational healthcare services predominantly from private companies (in 2020, for 86% of occupational health care recipients). [5] Occupational healthcare is funded by statutory payments collected from both employers and employees. However, in the Finnish context, occupational healthcare does not cover family members or extend to retirement.

Private healthcare offers primary care and a selection of specialist healthcare. Private healthcare is free-market-based and the prices are not regulated. Individuals can directly choose to visit specialists without gatekeeping. Private services are partly subsidised through the NHI scheme. The NHI reimbursements have gradually decreased over time and users bear most of the costs themselves. The popularity of voluntary private health insurance has been growing [6]. From 2012 to 2022 the uptake of private health insurance for adults grew 58%.

All permanent residents in Finland are entitled to *medicine reimbursements* for outpatient prescription medicines reimbursable from the NHI. While the clinical guidelines do not take economic aspects into account, the reimbursement decisions include pharmacoeconomic evaluations and budgetary considerations [7].

The universal basic reimbursement rate is 40% of the retail price. In addition, there are two other categories with a higher, disease-based special reimbursement rate (65% and 100%, the latter with a fixed fee of €4.50 per dispensing) for patients with certain chronic and severe diseases, including diabetes [8]. Antidiabetic medicines were included in the 100% category until 2017, when they were moved to the 65% category. Insulins remained reimbursed in the 100% category. Reimbursements for novel, typically more expensive, pharmaceuticals are often restricted to patients meeting specific clinical criteria. Eligibility to higher reimbursement and restricted reimbursements are granted based on a doctor's certificate. Since 2016, the reimbursement categories only apply after patients have met an initial deductible of €50 per calendar year (children and youth are exempt). An annual co-payment ceiling (€605.13 in 2018) protects patients from very high cumulative co-payment expenditure, though some co-payment (€2.50 per product per dispensing) applies even after meeting the annual ceiling. (See also, [9].)

References

1. Keskimaki I, Tynkkynen LK, Reissell E, Koivusalo M, Syrja V, Vuorenkoski L, Rechel B, Karanikolos M. Finland: Health System Review. Health Syst Transit. 2019 Aug;21(2):1-166.
2. Ministry of social affairs and health. Client charges in healthcare and social welfare. https://stm.fi/en/client-fees Accessed 12 Oct 2023.
3. Tervola J, Aaltonen K, Tallgren F. Can people afford to pay for health care? New evidence on financial protection in Finland. Copenhagen: WHO Regional Office for Europe; 2021. Licence: CCBY-NC-SA3.0 IGO.
4. Tynkkynen LK, Keskimäki I, Karanikolos M, Litvinova Y. Finland: Health system summary, 2023. 2023. ISSN 2958-9193 (online) ISBN 9789289059398. https://eurohealthobservatory.who.int/publications/i/finland-health-system-summary. Accesssed 31. Oct 2023.
5. Kela 2022. Kelan työterveyshuoltotilasto 2020. Helsinki; Kela; 2022
6. Finance Finland. Statistics on voluntary health insurance. 2023. https://www.finanssiala.fi/julkaisut/tilasto-sairauskuluvakuutus-2009-2022/ . Accessed 11 Oct 2023.
7. Health Insurance Act 1224/2004
8. Government Decree on the amendment of Government Decree on the diseases regarded as severe and chronic on medical grounds, and the medicinal costs for which a 65 or 100 per cent reimbursement is made under Chapter 5, section 5(2) of the Health Insurance Act. https://www.finlex.fi/fi/laki/kaannokset/2016/en20161149 Accessed 8 Nov 2024.
9. Kela. Reimbursements for medicine expenses. 2023. https://www.kela.fi/medicine-expenses. Accessed 11 Oct 2023.
