# Supplementary material for: Prescribing patterns before the initiation of novel antidiabetic medicines in public, occupational, and private healthcare: a register study reflecting the guidelines of care in type 2 diabetes
Source: BMC Health Serv Res. 2024 Dec 5;24:1553. doi: 10.1186/s12913-024-12010-y (PMC11619279; doi:10.1186/s12913-024-12010-y)
Supplement: Supplementary file 2 — Supplementary Material 2. [file 12913_2024_12010_MOESM2_ESM.pdf]

Supplementary file 2.

Rules used to link information on the health care service use to the prescription, adapted from Miettinen et al. 2016. The rules are executed in ascending order. Rules 6-12 use annual information on all prescriptions (not just information on prescriptions of antidiabetic medicines).

**Information used to link prescription to healthcare visit**	**Healthcare visit data used**	**Sectors the rule can link the prescription to**
1. Patient, physician, date	Public sector	Public sector
2. Patient, physician, date	Private sector	Private sector
3. Patient, date	Occupational sector	Occupational sector
4. Patient, date	Public sector	Public sector
5. Patient, date	Private sector	Private sector
6. Prior physician – patient - combination, if service use only in one sector	All sectors	Public sector, private sector
7. The patient had at least one visit in public sector, most of the patient's prescriptions were written in public sector, most of the physician's prescriptions were written in public sector	All sectors	Public sector
8. The patient had at least one visit in occupational sector, most of the patient's prescriptions were written in occupational sector	All sectors	Occupational sector
9. The patient had at least one visit in private sector, most of the patient's prescriptions were written in private sector, most of the physician's prescriptions were written in private sector	All sectors	Private sector
10. The patient had at least one visit in public sector	All sectors	Public sector
11. The patient had at least one visit in occupational sector	All sectors	Occupational sector
12. The patient had at least one visit in private sector	All sectors	Private sector
13. -		Public sector

Miettinen J, Aaltonen K, Martikainen J. Lääkemääräysten alkuperän määrittäminen terveydenhuollon käyntitietojen avulla. Työpapereita 108. Helsinki: Kela; 2016.
