# Supplementary material for: Prescribing patterns before the initiation of novel antidiabetic medicines in public, occupational, and private healthcare: a register study reflecting the guidelines of care in type 2 diabetes
Source: BMC Health Serv Res. 2024 Dec 5;24:1553. doi: 10.1186/s12913-024-12010-y (PMC11619279; doi:10.1186/s12913-024-12010-y)
Supplement: Supplementary file 3 — Supplementary Material 3. [file 12913_2024_12010_MOESM3_ESM.pdf]

Supplementary file 3.

Full models.

All initiators

	OR	95% CI		P	OR	95% CI		P
**Model 1: Prior use of another non-insulin antidiabetic**								
Occupational healthcare vs. public healthcare	1.274	0.739	2.197	0.3832	1.252	0.724	2.165	0.4221
Private healthcare vs. public healthcare	0.558	0.308	1.009	0.0535	0.511	0.281	0.927	0.0273
Income quartile II vs I	0.686	0.421	1.117	0.1293	0.686	0.419	1.121	0.1326
Income quartile III vs I	1.167	0.679	2.005	0.5761	1.163	0.675	2.004	0.5871
Income quartile IV vs I	0.764	0.443	1.319	0.3345	0.752	0.435	1.300	0.3074
Sex (Male vs Female)	1.303	0.915	1.855	0.1422	1.299	0.911	1.852	0.1487
Comorbidity (Yes vs No)	1.695	1.165	2.464	0.0058	1.596	1.093	2.330	0.0155
Diabetes-related hospital contact (Yes vs No)	0.415	0.251	0.686	0.0006	0.397	0.239	0.660	0.0004
Age	1.009	0.994	1.025	0.2347	1.012	0.997	1.028	0.1215
Initiation year 2015 vs 2014					1.135	0.337	3.824	0.8387
Initiation year 2016 vs 2014					0.620	0.235	1.637	0.3349
Initiation year 2017 vs 2014					0.416	0.161	1.076	0.0703
Initiation year 2018 vs 2014					0.325	0.126	0.837	0.0198
**Model 2: Prior use of statin**								
Occupational healthcare vs. public healthcare	0.817	0.628	1.062	0.1301	0.818	0.629	1.064	0.1350
Private healthcare vs. public healthcare	0.761	0.525	1.102	0.1481	0.741	0.511	1.076	0.1150
Income quartile II vs I	1.022	0.788	1.325	0.8708	1.011	0.779	1.312	0.9326
Income quartile III vs I	1.131	0.868	1.472	0.3614	1.117	0.858	1.456	0.4110
Income quartile IV vs I	0.905	0.680	1.205	0.4943	0.895	0.672	1.191	0.4460
Sex (Male vs Female)	1.583	1.316	1.904	<.0001	1.586	1.318	1.908	<.0001
Comorbidity (Yes vs No)	1.689	1.386	2.059	<.0001	1.673	1.372	2.041	<.0001
Diabetes-related hospital contact (Yes vs No)	0.903	0.642	1.272	0.5608	0.901	0.639	1.270	0.5518
Age	1.040	1.031	1.049	<.0001	1.041	1.032	1.050	<.0001
Initiation year 2015 vs 2014					0.766	0.485	1.210	0.2530
Initiation year 2016 vs 2014					0.700	0.474	1.034	0.0734
Initiation year 2017 vs 2014					0.778	0.526	1.152	0.2100
Initiation year 2018 vs 2014					0.684	0.461	1.016	0.0600
**Model 3: Prior use of insulin**								
Occupational healthcare vs. public healthcare	0.372	0.264	0.523	<.0001	0.345	0.244	0.487	<.0001
Private healthcare vs. public healthcare	0.838	0.563	1.248	0.3844	0.755	0.503	1.134	0.1758
Income quartile II vs I	0.943	0.731	1.216	0.6500	0.936	0.723	1.211	0.6128
Income quartile III vs I	0.827	0.634	1.079	0.1615	0.836	0.639	1.094	0.1914
Income quartile IV vs I	0.670	0.494	0.908	0.0100	0.661	0.485	0.900	0.0086
Sex (Male vs Female)	1.006	0.830	1.220	0.9501	1.006	0.828	1.223	0.9503
Comorbidity (Yes vs No)	1.398	1.115	1.753	0.0037	1.326	1.054	1.668	0.0158
Diabetes-related hospital contact (Yes vs No)	4.798	3.420	6.730	<.0001	4.777	3.390	6.732	<.0001
Age	0.994	0.986	1.003	0.1923	0.999	0.990	1.007	0.7431

Initiation year 2015 vs 2014	0.609	0.387	0.958	0.0319
Initiation year 2016 vs 2014	0.424	0.288	0.624	<.0001
Initiation year 2017 vs 2014	0.263	0.177	0.390	<.0001
Initiation year 2018 vs 2014	0.375	0.253	0.557	<.0001

Working-age initiators

	OR	95% CI		p-Value	OR	95% CI		p-Value
**Model 1: Prior use of another non-insulin antidiabetic**								
Occupational healthcare vs. public healthcare	1.121	0.609	2.061	0.7142	1.103	0.599	2.031	0.7525
Private healthcare vs. public healthcare	0.533	0.221	1.286	0.1613	0.481	0.197	1.173	0.1076
Income quartile II vs I	0.698	0.360	1.352	0.2865	0.681	0.351	1.324	0.2575
Income quartile III vs I	0.966	0.460	2.029	0.9273	0.961	0.456	2.025	0.9157
Income quartile IV vs I	0.767	0.355	1.655	0.4982	0.770	0.356	1.665	0.5066
Sex (Male vs Female)	1.457	0.907	2.340	0.1200	1.468	0.912	2.362	0.1137
Comorbidity (Yes vs No)	2.363	1.453	3.843	0.0005	2.228	1.402	3.732	0.0009
Diabetes-related hospital contact (Yes vs No)	0.880	0.364	2.132	0.7777	0.863	0.355	2.098	0.7443
Age	1.025	0.998	1.052	0.0683	1.025	0.998	1.053	0.0649
Initiation year 2015 vs 2014					0.809	0.208	3.141	0.7595
Initiation year 2016 vs 2014					0.549	0.181	1.665	0.2893
Initiation year 2017 vs 2014					0.433	0.145	1.290	0.1329
Initiation year 2018 vs 2014					0.411	0.137	1.232	0.1124
**Model 2: Prior use of statin**								
Occupational healthcare vs. public healthcare	0.781	0.581	1.049	0.1007	0.778	0.578	1.046	0.0967
Private healthcare vs. public healthcare	0.782	0.463	1.321	0.3583	0.760	0.447	1.289	0.3083
Income quartile II vs I	1.019	0.733	1.416	0.9123	1.009	0.725	1.403	0.9597
Income quartile III vs I	1.136	0.795	1.623	0.4850	1.130	0.790	1.617	0.5033
Income quartile IV vs I	0.902	0.620	1.313	0.5899	0.900	0.618	1.312	0.5851
Sex (Male vs Female)	1.482	1.170	1.878	0.0011	1.479	1.167	1.874	0.0012
Comorbidity (Yes vs No)	1.616	1.275	2.047	<.0001	1.600	1.261	2.030	0.0001
Diabetes-related hospital contact (Yes vs No)	1.187	0.746	1.887	0.4695	1.186	0.745	1.888	0.4730
Age	1.058	1.042	1.073	<.0001	1.058	1.042	1.074	<.0001
Initiation year 2015 vs 2014					0.771	0.456	1.305	0.3331
Initiation year 2016 vs 2014					0.826	0.527	1.295	0.4044
Initiation year 2017 vs 2014					0.748	0.475	1.175	0.2077
Initiation year 2018 vs 2014					0.702	0.444	1.112	0.1316
**Model 3: Prior use of insulin**								
Occupational healthcare vs. public healthcare	0.374	0.257	0.543	<.0001	0.334	0.228	0.490	<.0001
Private healthcare vs. public healthcare	0.721	0.396	1.314	0.2859	0.575	0.308	1.074	0.0825
Income quartile II vs I	0.989	0.705	1.389	0.9502	0.968	0.684	1.371	0.8560
Income quartile III vs I	0.785	0.533	1.156	0.2205	0.792	0.533	1.177	0.2482
Income quartile IV vs I	0.645	0.422	0.986	0.0429	0.653	0.423	1.006	0.0533
Sex (Male vs Female)	1.184	0.906	1.547	0.2156	1.182	0.899	1.553	0.2303
Comorbidity (Yes vs No)	1.110	0.843	1.463	0.4576	1.053	0.795	1.396	0.7174
Diabetes-related hospital contact (Yes vs No)	7.206	4.373	11.873	<.0001	7.276	4.374	12.102	<.0001
Age	0.998	0.982	1.014	0.8179	1.001	0.985	1.018	0.8813
Initiation year 2015 vs 2014					0.591	0.342	1.020	0.0587
Initiation year 2016 vs 2014					0.398	0.249	0.634	0.0001
Initiation year 2017 vs 2014					0.204	0.124	0.334	<.0001
Initiation year 2018 vs 2014					0.381	0.235	0.617	<.0001
